# Lessons from humanitarian clusters to strengthen health system responses to mass displacement in low and middle-income countries: A scoping review

**DOI:** 10.1016/j.jmh.2020.100028

**Published:** 2020-12-09

**Authors:** Anna Durrance-Bagale, Omar Mukhtar Salman, Maryam Omar, Mervat Alhaffar, Muhammad Ferdaus, Sanjida Newaz, Sneha Krishnan, Natasha Howard

**Affiliations:** a Department of Global Health and Development, London School of Hygiene and Tropical Medicine, 15-17 Tavistock Place, London WC1H 9SH, United Kingdom; b Bart's Health NHS Trust, The Royal London Hospital, Whitechapel Road, London E1 1BB, United Kingdom; c BRAC University, UB04 – 66 Bir Uttam AK Khandakar Road, Dhaka 1212, Bangladesh; d Department of Community Health Sciences, Rady Faculty of Health Sciences, University of Manitoba, 750 Bannatyne Ave, Winnipeg MB R3E 0W2, Canada; e Environment, Technology and Community Health (ETCH) Consultancy Services, Mumbai, India; f National University of Singapore, Saw Swee Hock School of Public Health, 12 Science Drive 2, 117549, Singapore

**Keywords:** Mass displacement, Humanitarian cluster, Health systems

## Abstract

The humanitarian cluster approach was established in 2005 but clarity on how lessons from humanitarian clusters can inform and strengthen health system responses to mass displacement in low and middle-income countries (LMIC) is lacking. We conducted a scoping review to examine the extent and nature of existing research and identify relevant lessons.

We used Arksey and O'Malley's scoping framework with Levac's 2010 revisions and Khalil's 2016 refinements, focussing on identifying lessons from discrete humanitarian clusters that could strengthen health system responses to mass population displacement. We summarised thematically by cluster.

Of 186 sources included, 56% were peer-reviewed research articles. Most related to health (37%), protection (18%), or nutrition (13%) clusters. Key lessons for health system responses included the necessity of empowering women; ensuring communities are engaged in decision-making processes (e.g. planning and construction of camps and housing) to strengthen trust and bonds between and within communities; and involving potential end-users in technological innovations development (e.g. geographical information systems) to ensure relevance and applicability.

Our review provided evidence that non-health clusters can contribute to improving health outcomes using focussed interventions for implementation by government or humanitarian partners to inform LMIC health system responses to mass displacement.

## Highlights

• Potential scope for health-system learning from cluster responses to mass displacement.
• Non-health clusters can contribute to health improvements during mass displacement.
• Cluster approaches are often siloed with insufficient cross-cluster learning.
• Equitable power dynamics between displaced people, humanitarian actors, and governments are still needed.

## Introduction

While humanitarian response coordination has developed significantly, sectors generally maintain siloed approaches, with minimal cross-sectoral engagement and learning (Moslehi et al*.*, 2016; Bealt and Mansouri, 2018). International humanitarian system reform in 2005 aimed to address these issues, introducing elements to enhance ‘predictability, accountability and partnership’ including the Cluster approach (United Nations Office, 2020). The Inter-Agency Standing Committee consolidated United Nations (UN) and humanitarian organisations within eleven clusters, with designated lead agencies for each, and clear roles and coordination responsibilities (United Nations High Commissioner for Refugees. Emergency Handbook 2016. Available from: https://emergency.unhcr.org/). This approach is enacted in humanitarian emergencies if national governments are unable/unwilling to provide humanitarian relief and international support is required.

The COVID-19 pandemic has highlighted that we need effective global health governance and multisectoral approaches that account for social determinants of health - where and how people live and issues that will affect their quality-of-life - and ensure migrant and displaced populations are not left behind (Gostin et al*.*, 2020).

Numbers of forcibly-displaced people fleeing conflict and disaster are increasing, with approximately 70.8 million people displaced globally: 41.3 million internally-displaced, 25.9 million classed as refugees, and 3.5 million designated as asylum-seekers (United Nations High Commissioner, 2020). Figures highlight the importance of humanitarian workers sharing experiences and learning across sectors to improve conditions during mass displacement (i.e. rapid forced displacement of large numbers of people, including in Bangladesh and Syria). To provide better health outcomes for displaced populations the system requires a coordinated response between clusters to strengthen the national health system response. Despite extensive literature on health interventions, we found very little on potential links between health and non-health clusters despite all supporting displaced populations (Blanchet et al*.*, 2017).

We thus conducted a scoping review of peer-reviewed literature on responses of the eleven humanitarian clusters – i.e. Education (Unicef/Save the Children), Early Recovery (UNDP), Food Security (WFP/FAO), Health (WHO), Nutrition (Unicef), Protection (UNHCR), Shelter (IFRC/UNHCR), Water, Sanitation and Hygiene (Unicef), Camp Coordination and Management (IOM/UNHCR), Emergency Telecommunication (WFP), and Logistics (WFP) – to mass displacement in low and middle-income countries (LMICs). Objectives were to: (i) summarise the scope of the literature on humanitarian cluster interventions responding to mass displacement; and (ii) identify lessons from cluster interventions of use in strengthening health system responses to mass displacement.

## Methods

### Study design

We conducted a scoping review using Arksey and O'Malley's scoping framework with Levac et al.’s 2010 revisions and Khalil et al*.*’s 2016 refinements (Woodward et al*.*, 2014; Khalil et al*.*, 2016; Peters et al*.*, 2015; Arksey and O'Malley, 2005; Levac et al*.*, 2010). Table 1 shows definitions used. Table 2 shows 98 countries eligible for inclusion. We identified 100 countries and territories with international humanitarian cluster responses to address mass displacement between 2005 and 2019, based on World Bank and United Nations Office for the Coordination of Humanitarian Affairs (UNOCHA) Financial Tracking Service criteria, and thus eligible for inclusion. We excluded high-income countries using World Bank criteria (i.e. Israel, USA).

**Table 1:** Definitions.

Cluster	A group of agencies working together toward common objectives within an emergency response sector. The cluster approach, instituted in 2006 as part of UN Humanitarian Reform, includes 11 global clusters. (World Health Organization, 2020)
Health system	The people, institutions and resources, arranged together in accordance with established policies to improve the health of the population they serve, while responding to people's legitimate expectations and protecting them against the cost of ill-health through a variety of activities whose primary intent is to improve health. (World Health Organization, 2020)
Humanitarian emergency/ crisis	Humanitarian action occurs in “a range of situations including natural disasters, conflict, slow and rapid-onset events, rural and urban environments, and complex political emergencies in all countries.” (SPHERE. SPHERE Glossary, 2019)
Humanitarian setting	National and subnational settings experiencing a ‘humanitarian emergency’, for which a humanitarian response has been mobilised, including any phase of the emergency or recovery process.
Internally-displaced person (IDPs)	"Persons or groups of persons who have been forced or obliged to leave their homes or places of habitual residence, in particular as a result of or to avoid the effects of armed conflict, situations of generalized violence, violations of human rights or natural or human-made disasters, and who have not crossed an internationally-recognized state border." (United Nations High Commissioner for Refugees. Emergency Handbook 2016. Available from: https://emergency.unhcr.org/)
Low and middle-income	Low-income economies are those with a GNI per capita of US$995 or less in 2017; lower middle-income economies have a GNI per capita of US$996-US$3895; upper middle-income economies have a GNI per capita of US$3896-US$12,055. (World Bank, 2019)
Mass displacement	The sudden displacement of a large number of people (Kirbyshire et al*.*, 2017), who are forced to leave their homes, either within or across national borders, owing to sudden shocks or stresses, including armed conflict, civil unrest, or natural or man-made disasters.
Refugee	A person forced to flee their country of origin and who has a well-founded fear of returning. A refugee is typically acknowledged by UNHCR mandate, host nation, and international and regional treaties. (United Nations High Commissioner for Refugees, 2005)

**Table 2:** Countries/territories affected by mass displacement generating an international humanitarian response (UNOCHA 2005–2019).

Country	UNOCHA	Country	UNOCHA
Afghanistan	2009–2019	Lesotho	2007, 2012, 2013
Albania	2017	Liberia	2006–2008, 2012
Algeria	2018–2019	Libya	2011, 2014–2018
Angola	2005	Malaysia	2018
Azerbaijan	2019	Malawi	2019
Bangladesh	2015–2019	Mali	2012–2019
Benin	2005	Mauritania	2012–2016
Bolivia	2007–2008	Madagascar	2007–2019
Bosnia & Herzegovina	2018–2019	Marshall Islands	2015
Brazil	2019	Micronesia	2019
Burkina Faso	2007, 2009, 2012–2017	Mongolia	2010
Burundi	2005–2019	Mozambique	2007, 2017- 2019
Cambodia	2014–2019	Myanmar	2008–2019
Cameroon	2014–2019	Namibia	2009, 2011
Central African Republic	2005–2019	Nepal	2006
Chechnya and neighbours	2005	Nicaragua	2007, 2011, 2015
Chad	2005–2019	Niger	2005, 2012, 2019
Colombia	2019	Nigeria	2014–2019
Congo, Republic of	2014, 2018	Pakistan	2007–2011
Cote d'Ivoire	2005–2006, 2007, 2012	Palestine, Occupied (West Bank & Gaza)	2006–2019
Dominica	2017	Papua New Guinea	2019
Dominican Republic	2007	Peru	2007, 2017, 2019
DRC	2005–2019	Philippines	2009–2014
Djibouti	2005, 2011–2017	Rwanda	2019
Ecuador	2016, 2019	Sao Tome and Principe	2008
Egypt	2016–2019	Senegal	2014–2017
El Salvador	2009–2012	Sierra Leone	2008
Eritrea	2005	Solomon Islands	2019
Ethiopia	2017–2019	Somalia	2005–2019
Fiji	2016	South Sudan	2011–2019
Gambia, The	2014–2016	Sri Lanka	2011
Georgia	2008	Sudan	2005–2019
Ghana	2007	Swaziland	2007
Guyana	2005	Syria	2012–2019
Guatemala	2005, 2010, 2016	Tajikistan	2008
Guinea	2005	Thailand	2018–2019
Guinea-Bissau	2006	Timor-Leste	2006–2007
Haiti	2008, 2010–2019	Tonga	2018
Honduras	2015–2016	Turkey	2016–2019
Indonesia	2006, 2009	Tunisia	2019
Iran	2019	Tuvalu	2015
Iraq	2008–2019	Uganda	2005–2010
Jordan*	2016–2019	Ukraine	2014–2019
Kenya	2006, 2008–2017	Uzbekistan	2019
Korea DPR	2007	Vanuatu	2015
Kosovo	2017	Western Sahara	2016
Kyrgyzstan	2008, 2010	Yemen	2008–2019
Lao	2009	Zambia	2007
Lebanon	2006	Zimbabwe	2006–2012, 2016, 2019

#### Defining the research question

We selected: “What lessons from humanitarian cluster responses can be applied to strengthening health system responses to mass population displacement in LMICs?”

#### Identifying relevant sources

Table 3 shows the five databases OMS and ADB systematically searched (October 2019) and eight websites SK, OMS, ADB and MF purposively searched (April 2020). Additionally, ADB purposively searched reference lists of all included literature (April 2020).

**Table 3:** Databases and websites included.

Databases	Websites
Embase	ALNAP
Global Health	Google and Google Scholar (first 100 results each)
Medline	3ie impact evaluations database
PsycINFO	IOM (International Organisation for Migration)
Web of Science	IPA (Innovations for Poverty Action)
	ELDIS (Institute of Development Studies)
	Shelter Cluster

OMS and ADB used relevant terminology for ‘Health cluster/sector’ OR ‘Education cluster/sector’ OR ‘Early Recovery cluster/sector’ OR ‘Shelter cluster/sector’ OR ‘Food Security cluster/sector’ OR ‘Nutrition cluster/sector’ OR ‘Protection cluster/sector’ OR ‘Water, Sanitation and Hygiene cluster/sector’ OR ‘Camp and coordination and management cluster/sector’ OR ‘Emergency telecommunications cluster/sector’ OR ‘Logistics cluster/sector’ AND ‘humanitarian emergency’ AND ‘displaced populations’ AND eligible countries, adapted to subject headings for each database. SK, OMS, ADB and MF used similar search terms, e.g. ‘cluster’, ‘humanitarian’, ‘displacement’ and ‘recovery’, to mine grey literature.

#### Selecting sources

All authors agreed eligibility criteria iteratively, from initial criteria based on the research question and research data sources (World Bank, 2019). Table 4 provides final eligibility criteria. OMS, MA and ADB removed duplicates using Mendeley Desktop v1.19.4. OMS, ADB, SK, MO and MA screened titles and abstracts against eligibility criteria using Covidence software (www.covidence.org). ADB, SK, MO, MA, OMS and NH double-screened full-texts against eligibility criteria.

**Table 4:** Eligibility criteria.

Category	Inclusion criteria	Exclusion criteria
Context	• Humanitarian settings, affected by mass displacement, as per study definition.	• Other settings.
Humanitarian phase	• Peri/post-emergency, including relief, recovery and reconstruction phases.	• Pre-emergency, e.g. preparedness and mitigation.
Topic	• Education cluster or domain. • Food security cluster or domain. • Health cluster or domain. • Early recovery cluster/domain, including post-disaster recovery programmes. • Nutrition cluster or domain. • Protection cluster or domain. • Shelter cluster/domain, including post-disaster reconstruction and recovery programmes. • WASH cluster or domain. • Coordination and management domain. • Communications domain. • Logistics domain.	• Other sectors.
Source type	• Original empirical evidence related to humanitarian clusters/sectors responding to mass displacement, including: • Research articles. • Systematic/scoping reviews. • Conference abstracts. • Book chapters. • Agency technical reports.	• Conference abstracts covering material in a publication. • Case studies on individual or household. • Protocols, methods description only. • Audio/video reports, blog posts. • Social media/media articles. • Guidance documents.
Study design	• All study designs	• No research component. • Entirely theoretical.
Participants/ population of interest	• Populations in humanitarian settings affected by mass displacement, including internally-displaced and refugees. • All genders and ages.	• Humanitarian settings with no displacement. • Emergency settings with no humanitarian cluster response.
Time-period	• Retrospective and prospective.	• Future.
Publication year	• 2005 onward.	• Pre-2005.
Publication Language	• Arabic, Chinese, English, French, Russian, Spanish, Hindi.	• Sources in other languages.

#### Charting data

ADB, SK, OMS, MO, MA, and SN extracted data to an Excel sheet using the following headings:

• source identifiers, i.e. publication year, lead author, source type, search type/name;
• source characteristics, i.e. country, study design, data collection, time-period, primary methods, participant numbers/characteristics.
• main findings (Quantitative: measures of effect or impact reported, primary outcomes; Qualitative: quotations, related narrative excerpts).

#### Synthesising and reporting

All authors summarised the number of sources by where accessed, type, distribution, and nature. ADB, SK, MO, MA, OMS, SN and NH summarised data thematically using a narrative synthesis approach (Popay et al*.*, 2006) and identified implications for policy, practice, and research.

## Results

### Extent and nature of literature

**Fig. 1:**
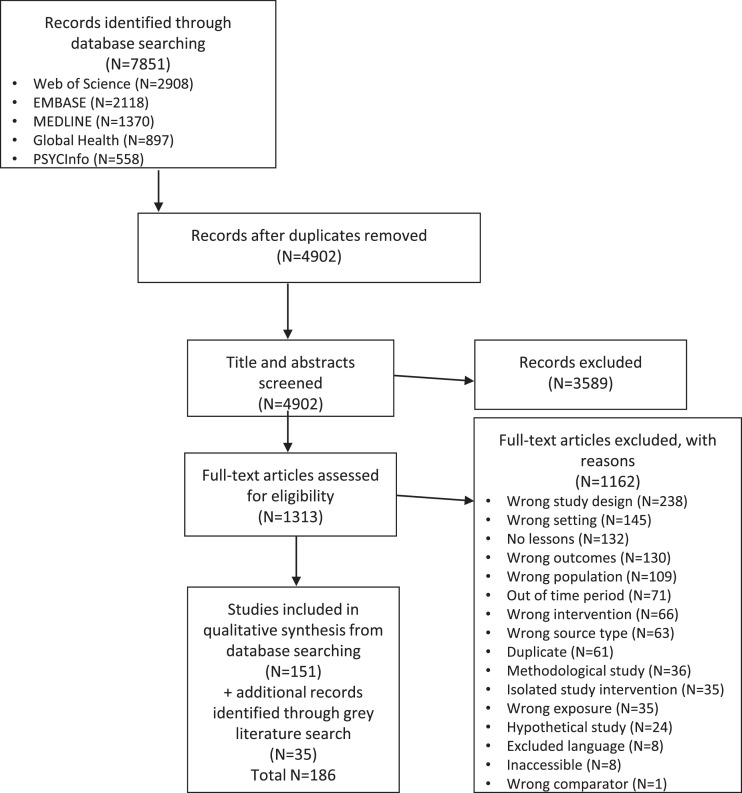
Flow diagram.

Fig. 1 shows 7851 documents identified, 2949 excluded as duplicates, 3589 excluded through title/abstract screening, 1162 excluded through full-text screening, and 151 included along with 35 eligible hand-searched documents. Thus, 186 documents were included.

Publications were from public health, anthropology, policy and epidemiology disciplines, including 105 (56%) peer-reviewed research articles, 27 (15%) opinion articles, 49 (26%) agency reports, three (2%) dissertations, one (<1%) conference abstract, and one (<1%) website report. Methods and study designs were often unclear - among 153 sources, 42 (23%) were qualitative, 27 (15%) were epidemiological observational studies, 24 (13%) used mixed-methods, and nine (5%) were trials. Classified by main sector, 68 (37%) were health, 33 (18%) protection, 25 (13%) nutrition, 17 (9%) shelter, 11 (6%) water, sanitation and hygiene (WASH), 11 (6%) coordination/management, 9 (5%) logistics, 8 (4%) education, 2 (1%) telecommunications, 1 (<1%) early recovery, and 1 (<1%) food security.

### Health system lessons

Table 5 provides major lessons from the 11 clusters and how these might inform health system responses to mass displacement.

**Table 5:** Key lessons by cluster.

Sector (source numbers)	Lessons
Health (68)	Non-communicable disease and mental health programmes could be integrated.
	Trained CHWs could implement mental health programmes, focusing on identifying most severe or acute cases.
	Education and empowerment for adolescent female refugees are essential.
	Cash-transfers, either unconditional or conditional, can promote better health.
Protection (33)	Women's groups should be promoted and empowered in displacement settings, as should similar groups for adolescents
	Mobile health services could support dispersed displaced communities (i.e. those not in camps).
Nutrition (25)	Provide recovery interventions, e.g. baby tents and targeted support, to caregivers of malnourished children.
	Strengthen capacity of existing health facilities through providing supplies and equipment, training staff, and regular support/monitoring.
	Planning for cluster transitioning should begin as early as possible to restore adequate and sustainable coordination mechanisms, focusing on supporting government functions and capacity in emergency nutrition response.
	Cash-transfers can strengthen host country economies, particularly where logistics for in-kind humanitarian distributions are expensive and complex.
Shelter (17)	Ensure host and displaced communities are involved in camp planning to encourage trust and reduce resentment over resource allocation.
Coordination/ management (11)	Drones could be used to estimate displaced population sizes and locations.
	Camp managers should create an enabling environment that normalises employment opportunities for displaced people.
WASH (11)	Install latrine blocks for specified cabins, each connected to a biogas plant and kitchen, with space to dry menstrual hygiene items and one latrine unit per block designed as child-friendly.
	Supertowel could be trialled in displacement settings to examine usage barriers.
Logistics (9)	GIS can improve responses and effective community engagement, e.g. by locating populations, particularly in hard-to-reach areas.
	Rapid assessments should include socio-cultural context to inform humanitarian healthcare approaches.
	Build capacity through supporting national NGO implementation.
Education (8)	Peer education can benefit trainers and trainees and works for both adults and children, particularly interactive sessions.
	Community-based activities should involve those with local/contextual understanding, particularly respected community leaders.
	Use networks of organisations and ensure displaced people are included in project design and implementation.
Telecommunications (2)	Technology should involve potential end-users in the design phase to ensure applicability and relevance.
Food security (1)	Voucher programmes could help link displaced and host communities and support local economies.
Early recovery (1)	Need for preparation rather than short-term response initiatives.

### Health

#### Mental health

Seventeen health-sector sources discussed mental health, primarily the importance of interventions for refugees and internally-displaced people (IDPs). Primary care physicians in Pakistan were often unable to recognise or effectively manage mental health issues in these populations (Humayun et al*.*, 2017). Interventions that created strong networks of community health-workers (CHWs) improved follow-up and treatment adherence in the Philippines (Mueller et al*.*, 2011). Similarly, a psychiatric liaison service at the main casualty hospital and weekly, mobile mental health clinics provided flexibility in Haiti (Rose et al*.*, 2011). General practitioners and psychosocial workers can receive on-the-job training and supervision from specialists. Trained psychiatric nurses can provide supervision in refugee health facilities, supported by regular visits by a psychiatrist and supplemental (phone calls/messaging) support (Echeverri et al*.*, 2018).

Store-and-forward tele-mental health used indirect transmission of electronic text or recorded audio-visuals to provide training for health-workers in Syria (Jefee-Bahloul et al*.*, 2016). However, tele-mental health acceptability in terms of patient concerns over security and stigma in such challenging circumstances remains questionable (Jefee-Bahloul et al*.*, 2016). Teaching recovery techniques to parents may both reduce children's post-traumatic stress and increase caregiver self-efficacy and use of effective strategies (El-Khani et al*.*, 2018). James proposed a culturally-sensitive mental health intervention using a psychological framework to benefit participants without disrupting access for pre-existing communities in Haiti (James, 2012). Involving survivors in critical service provision aimed to empower them by enhancing perceived social belonging, and influence over community-building outcomes.

Denying refugees employment affects dignity and mental wellbeing. If refugees are unemployed long-term, dependant on external aid, or unable to participate in social structures, they can develop psychosocial and health problems, as shown in Jordan (Schön et al*.*, 2018). Awareness raising of the importance of incorporating mental health and psychosocial support into other sectors is essential for integrated responses (Eloul et al*.*, 2013). Assessing cost-effectiveness of mental health interventions is essential as this dominates donor agendas (Abbara et al*.*, 2016).

#### Sexual and reproductive health (SRH)

Eleven sources discussed SRH importance during mass displacement. **Communication and education** campaigns should be run by people familiar with cultural traditions, languages and dialects, and preferably from the same community. Raising awareness and promoting SRH care services can be achieved using campaigns mobilising CHWs to disseminate information during home visits, clinic sessions, and public meetings, as shown in Sudan (Adam et al*.*, 2015). Similarly, self-esteem projects among adolescents may reduce HIV risk factors, including adolescent marriage and age-disparate, unprotected, and transactional sex (Bermudez et al*.*, 2019). As transactional sex is a reality in displacement settings, **economic initiatives** (e.g. cash transfers, increased income-generating activities) reduced it among adolescent girls in Ethiopia (Bermudez et al*.*, 2019).

While **family planning** services for displaced populations must include long-acting methods, provider training and infrastructure requirements to insert intra-uterine devices and implants are significantly greater than for dispensing pills or injectables (Curry et al*.*, 2015).

Duclos and colleagues emphasised that increasing the capacity of local health-workers to assist populations with **maternal and neonatal health** interventions in places where international staff are unable or unwilling to work can reinforce existing structures, as shown in Syria (Duclos et al*.*, 2019). Appropriate timing and level of service delivery helps ensure essential neonatal intervention packages address major causes of neonatal death in South Sudan (Sami et al*.*, 2018). Home visits in the first week of life can significantly reduce neonatal mortality, but can be difficult in camps (Sami et al*.*, 2018). Relatively brief group interventions in Uganda improved maternal involvement and reduced sadness/worry among displaced mothers of malnourished children. This involved mothers attending mother and baby groups and receiving a home visit, alongside a community-based therapeutic feeding programme in their displacement camp (Morris et al*.*, 2012).

#### Infectious and vaccine-preventable diseases

Fourteen sources discussed infectious diseases. Investing in national sentinel site **surveillance systems** to enable reporting of new cases and outbreaks is crucial (Magloire et al*.*, 2010). Improving humanitarian responses requires development and distribution of surveillance tools, an interdisciplinary strategy for rapid and reliable population census, and communication strategies using cellular networks, as shown in Haiti (Magloire et al*.*, 2010). Establishing surveillance systems, staffed with well-trained personnel, available vaccines and supplementary supplies, mobilisation of target populations, funding, coordination, access, security, and politics must be considered in delivering vaccination in humanitarian settings (Dasaklis and Pappis, 2017). Transmission dynamic models and forecasting can support outbreak epidemiology in displaced populations in Bangladesh, which is key to any successful intervention (Finger et al*.*, 2019). **Social mobilisation** is a key strategy to encourage vaccine uptake and address participation barriers, e.g. religious concerns, safety concerns, and perceived lack of sensitivity to gender norms including female vaccinators for girls (Jalloh et al*.*, 2020).

A source integrating vaccination and nutrition services among IDP children in South Sudan found that vaccinating at Outpatient Therapeutic Program (OTP) centres increased coverage, with dropout rates significantly lower among children vaccinated at OTP centres than at primary healthcare centres (Oladeji et al*.*, 2019). Successful vaccination during displacement requires strong leadership from existing coordination structures and must relate to long-term commitments to improve WASH indicators (Abubakar et al*.*, 2015). Curry and colleagues suggest increasing clinical skills training and supervision capacity to improve sustainability after donor-funded projects end (Curry et al*.*, 2015).

A mobile radiograph vehicle working with national **tuberculosis** programmes may increase uptake of chest radiographs for suspected cases of tuberculosis, as shown for Syrian refugees in Jordan (Cookson et al*.*, 2015), with similar effects through the use of standardised symptom screening at borders or during camp registration (Boyd and Cookson, 2019). Treatment success may be increased by planning for treatment continuation during emergencies. For example, Médecins Sans Frontières proposed a strategy for the evacuation of staff and the community that included providing ‘runaway bags’ with a supply of tuberculosis medications for their patients in Syria (Boyd and Cookson, 2019) – this idea is applicable to other illnesses with a significant medication burden, for example diabetes.

#### Non-communicable diseases (NCDs)

Five sources discussed NCDs. Pre-prepared, packaged food with low nutritional value, limited space for free movement, and lack of physical activity in the camps in the Philippines increases vulnerability to non-communicable diseases (Salazar et al*.*, 2018). **eHealth** strategies, especially the use of short message service (SMS) messages, could be easily implemented and adapted to suit most patients being treated for non-communicable disease. This low-cost, simple technique can be integrated into routine care as appointment reminders to improve compliance, daily reminders to take medications, and can help to raise awareness and overcome financial and geographical barriers. Primary care providers should be trained on using eHealth tools to increase awareness about diseases and encourage treatment compliance (Saleh et al*.*, 2018).

#### Integration

Three sources focused on integration. Expanding integrated community case management to include rapid malaria diagnostic tests, deworming kits, and vitamin A supplementation should be considered, as shown in South Sudan (Kozuki et al*.*, 2018). Using balanced scorecard approaches for monitoring and evaluating healthcare provision allows comparison across sites over time, potentially increasing equitable resource distribution (Malik, 2015). These involve developing indicators to describe and improve overall performance across four domains: (i) health-worker training; (ii) facility resources; (iii) community satisfaction and outreach; and (iv) service provision (Chan et al*.*, 2010).

### Protection

A major lesson from four protection sources is that displaced populations are at increased risk of sexual and gender-based violence (GBV), primarily affecting women and girls (Vu et al*.*, 2016, 2017; Stark et al*.*, 2018, 2017). Although GBV-specific services are available in many humanitarian settings, underreporting and underuse is common. GBV takes many forms and is associated with long-term health consequences including poor sexual and SRH, increased risk of infections, physical injury and disability, and poor mental health including suicidal ideation. Free, flexible GBV service delivery in women's own communities, e.g. mobile response and mitigation services, may reduce access barriers, including transportation, checkpoints, and domestic expectations, as shown in Colombia, Ethiopia, and Kenya (Vu et al*.*, 2016, 2017). Mobile services can additionally promote health-seeking behaviour, and offer services (e.g. screening, therapy, pharmacy) to reach women and other groups who cannot readily attend clinics, helping overcome cultural and physical barriers. As women are key in community mobilisation and conflict resolution (De La Puente, 2011), promoting women's involvement and status in society increases their security and ability to discuss their needs, as shown in Sudan. Humanitarian service providers can support this by recognising women as active community development partners (De La Puente, 2011), e.g. having women's groups - comprising displaced and host-community participants - discuss health issues in displaced communities. This could also act to encourage community cohesion and acceptance of refugees.

Three sources discussed child protection, indicating fostering provides better care for unaccompanied and separated children compared with residential care, shelters, or supported living. Best practice evidence suggests that foster families should have similar religious, culture-linguistic and ethnic characteristics to foster-children, as shown among Syrian refugees in Jordan (Suleiman AlMakhamreh and Hutchinson, 2018). Children's ideas of protection and how they protect themselves must be investigated, and power relations addressed (Bermudez et al*.*, 2019; Clark-Kazak, 2010).

Most IDPs in Colombia moved from rural to urban environments, a change with profound humanitarian and socioeconomic consequences (Carrillo, 2010), including efficient land use; overcrowding, e.g. due to rapid influxes; availability of government services; and integration of displaced populations into economies that may have limited employment opportunities (Carrillo, 2010).

### Nutrition

The nutrition sector provides examples of well-coordinated response plans in South Sudan (Ndungu and Tanaka, 2017), successes and challenges in community-based treatment of acute malnutrition in Uganda (Olu et al*.*, 2015), and capacity development of multiple stakeholders in addressing malnutrition in humanitarian emergencies in Kenya (Mwendwa et al*.*, 2016). Major topics included technical aspects of breastfeeding and complementary feeding (Shaker-Berbari et al*.*, 2018; Ayoya et al*.*, 2013; Abu-Taleb, 2015; Dolan et al*.*, 2015; Fänder and Frega, 2015), capacity-building to detect acute malnutrition, benefits of providing food parcels while transitioning from provision of food aid to cash inputs, unconditional cash transfers, baby tents in Haiti (Ayoya et al*.*, 2013), outpatient therapeutic programme discharge packages ensuring food security for at least 3 months in Somalia (Desie, 2017), and Super Cereal Plus for targeted supplementary feeding in Syria (Dolan et al*.*, 2015). Establishment of facility-based surveillance is essential to strengthening primary healthcare and collecting growth monitoring data. Providing primary health facility-level trainings in treating acute malnutrition helps humanitarian health preparedness and response. Optimal mother, infant and young child nutrition practices, especially early-initiation of exclusive breastfeeding and appropriate complementary feeding, should be encouraged in all sectors (Mwendwa et al*.*, 2016).

Cash-based assistance to displaced populations in Lebanon can increase consumption of essential items, e.g. food and cooking fuel, providing better nutrition and less reliance on ineffective coping strategies to meet food needs (Emergency Nutrition Network, 2018). Cash-transfers can provide effective assistance in functioning markets in which essentials are readily available (Emergency Nutrition Network, 2018). Host governments and humanitarian partners can contribute cash assistance instead of in-kind food donations (Inglis and Vargas, 2015). Cash-transfers may decrease financial burdens on displaced populations, though uncertainty remains around what happens once they are discontinued and cash-for-work project implementation can be laborious. Cash beneficiaries were reportedly happier than non-beneficiaries and more able to meet basic household needs, but reported greater stress - potentially as a consequence of cash-aid dependency and lack of control over when it could be discontinued (Emergency Nutrition Network, 2018). Frequent payments can be too managerially onerous during emergency onset, with weekly or less-frequent more manageable (Doocy et al*.*, 2006).

### Shelter

Agblorti & Grant articulated common shelter-related integration barriers, describing South Sudanese refugees in Ghana (Agblorti and Grant, 2019). First, land acquisition negotiations for refugee camps - in four of five camps - were fraught with mistrust and host nationals saying appropriate compensation was not paid. Second, stakeholder resource allocations - especially among humanitarians - address refugees and can increase host community resentment. To be equitable and effective, refugee assistance must also support host populations. Third, host-refugee interactions cut across many unacknowledged dimensions (Agblorti and Grant, 2019).

Hallak et al*.* described a goal programming model with multiple objectives comprising capacitated maximum covering, fixed-charge costs, and humanitarian considerations in Syria (Hallak et al*.*, 2019). Criteria included: (i) vulnerability, e.g. numbers with disabilities, numbers of pregnant/lactating women; (ii) economic factors, e.g. cash-for-work to repair and make shelters habitable; (iii) portable WASH facilities; and (iv) scalability (Hallak et al*.*, 2019). Authors outlined a useful potential framework for health-system responses, incorporating differing stakeholder needs and ‘beneficiary’ representatives’ identifying problems (Hallak et al*.*, 2019). Four essential components to successful reconstruction projects: (i) immediate reconstruction response; (ii) engaging local communities in rebuilding; (iii) creating permanent solutions; and (iv) mitigating future disasters. Incorporating local design and architecture in reconstruction or building of new health facilities could be an empowering, collaborative, and socially equitable development process, valuing and forging synergistic partnerships rooted in community priorities (Hallak et al*.*, 2019).

### WASH

WASH sources discussed handwashing challenges in displacement and approaches to water safety. Two focussed on handwashing with soap and Supertowel for displaced populations from Iraq and Eritrea (Watson et al*.*, 2019; White et al*.*, 2019). Barriers included insufficient access to water and soap, lack of social support for handwashing, and absence of handwashing facilities (Smadi et al*.*, 2018).

Harding and Kosonen discussed approaches to identifying requirements during displacement, including Lot Quality Assurance Sampling in Batil refugee camp, South Sudan (Harding et al*.*, 2017; Kosonen et al*.*, 2019). Factors associated with reduced likelihood of contaminated stored drinking water included obtaining water from improved sources, using chlorine solution, and free chlorine in stored water (Gupta et al*.*, 2007). Hygiene promotion that does not rely on trained health-workers repeatedly delivering messages may be effective. ‘Hardware’ interventions should not be separated from ‘software’/behaviour-change components, which has often happened in handwashing interventions, particularly in emergencies (Watson et al*.*, 2019). To improve water safety guidelines, strengthened coordination, intersectoral collaboration, and institutional and technical capacity for monitoring, evaluation, and enforcement of appropriate legislation, are crucial (Chanda et al*.*, 2013).

### Coordination/management

Sources proposed multiple innovative approaches. Magloire et al*.* described surveillance monitoring of IDP challenges in Haiti, which aimed to address rapid NGO staff and health-worker turnover, wide IDP geographic spread and high mobility, inadequate infrastructure, and lack of denominator information (Magloire et al*.*, 2010). Institutional partnering (e.g. US-CDC and NGOs) can improve implementation, e.g. in developing standard operating procedures and easy reliable tools, and distributing guidelines to improve system performance (Magloire et al*.*, 2010).

Other innovations included satellite and drone imagery to estimate displaced populations (Checchi et al*.*, 2013), a bottom-up approach using fieldworker mobile phones to collect rapid data on events onset in Kenya (Limbu et al*.*, 2015), and a Trackpad system to control essential items distribution in a Salvation Army-run camp in Port-au-Prince (Ergun et al*.*, 2014). Ergun et al*.* described how each household received a barcoded card, which NGO staff tracked during distributions to update a simple database (Ergun et al*.*, 2014).

### Logistics

Shaikh described benefits of GIS mapping for planning and delivering preventive and therapeutic SRH services to women in Somalia (Shaikh, 2008). GIS methods can contribute to rapid health needs assessments for service planning and delivery, e.g. in estimating affected areas, identifying hard-to-reach populations, mapping secure travel and entry points for service delivery (Shaikh, 2008).

Al-Adem et al*.* described international NGOs sponsoring national NGO partners, which employed national and ‘beneficiary’ staff, as increasing project security, effectiveness, and community accessibility in Jordan (Al Adem et al*.*, 2018). They noted the lack of financial trust between international and national NGOs as a frequent capacity-building barrier, as resource sharing (e.g. knowledge, skills, funds) would facilitate effective coordination and refugee support (Al Adem et al*.*, 2018). Implementation through national NGOs can provide better and more acceptable service delivery for displaced population (Al Adem et al*.*, 2018; Parkinson and Behrouzan, 2015). Recognising bureaucratic uncertainties, social resources, and lived experiences of displacement is important to understanding how healthcare services fit within wider displacement experience (Parkinson and Behrouzan, 2015).

### Education

Two sources on Syrian refugees found peer-education could be cost-effective within a strong partnership between humanitarian actors and national NGOs (Deane, May 2020; Karam et al*.*, 2017), supporting the mental health of peer educators and benefitting those being educated. Non-formal education (NFE) can help traumatised children and may be easier to organise than integration into mainstream schooling (Karam et al*.*, 2017). These programmes integrate art, play, music, storytelling, or sport into conventional numeracy, literacy, and language classes. Instructors are usually refugees themselves and children may find it easier to relate to them than to other teachers. These programmes, particularly when aligned with national educational policies and curricula, can bridge learning gaps and help displaced children later reintegrate into formal education, either on return or in host societies (Deane, May 2020; Karam et al*.*, 2017; Mendenhall, 2014).

Education findings that could inform health-system responses include: (i) training refugees/IDPs to train others, as people from within displaced communities can be better understood and acceptable; (ii) NFE could disseminate health information, e.g. handwashing, particularly through interactive drama/acting sessions for those who cannot read; and (iii) educational establishments should involve local communities in educating displaced adults and children, to increase the relevance of curricula. Sources indicated that support from communities who understand context and complexities is crucial to intervention success. Integrating displaced and host communities can increase acceptance and promote effective relationship-building (Ensign, 2016).

### Telecommunications

Robehmed proposed Iraqi users be involved in technology design phases to ensure relevance to their needs (Robehmed, 2019). Moorthy et al*.* advocated that communication be prioritised during emergencies in Asia and involve different interactions across humanitarian agencies, first responders, and support staff (Moorthy et al*.*, 2018).

### Food security

Luce found that voucher programmes helped introduce a sense of normalcy and built links between Syrian refugees and Jordanian host communities while supporting local economies, potentially promoting refugees’ acceptance in host communities (Luce, 2014). Chkam found camp congestion contributed to a nutritional emergency and epidemic in Kenya (Chkam, 2016). Olivius argued that participation of diverse stakeholders, including women, is important in agenda setting and programme implementation (Olivius, 2013).

### Early recovery

Sadiq et al*.*, addressing early recovery in Bangladesh, argued that short-term initiatives are not as effective as addressing pre-emergency vulnerabilities and long-term planning is essential for post-emergency building back (Sadik et al*.*, 2018).

## Discussion

This review synthesised humanitarian cluster initiatives with relevance for health system responses and highlights potential scope for health-system learning from cluster responses to humanitarian emergencies involving mass displacement. Non-health humanitarian clusters can contribute to improving health outcomes during mass displacement, particularly Education, Nutrition, Protection, Shelter, and WASH, but approaches are often siloed with insufficient cross-cluster learning. While many displacement challenges are cluster-specific, others are broader. For example, high staff turnover can undermine implementation, geographical spread of camps and settlements may challenge services provision or relations with host communities, while infrastructure damage may disrupt communication and services provision to both displaced and host communities (Magloire et al*.*, 2010; Carrillo, 2010; Agblorti and Grant, 2019; Parkinson and Behrouzan, 2015; Ensign, 2016). Other challenges are particularly relevant to displacement, including camp conditions, insecurity, constrained employment opportunities, and uncertainties about the future (Schön et al*.*, 2018; Carrillo, 2010).

Power dynamics between displaced people, humanitarian organisations, and host or origin governments need to be examined and addressed to strengthen any intervention (Clark-Kazak, 2010). Many sources emphasised the importance of ensuring displaced people could participate in designing and delivering interventions (James, 2012; Ensign, 2016; Robehmed, 2019; Olivius, 2013). For example, Olivius explored how participatory community-based approaches could promote gender equity (Olivius, 2013). However, debate continues on the extent and means of displaced people's participation in agenda-setting and project implementation (Olivius, 2013; Olivius, 2014).

The Grand Bargain is a commitment by donors to provide aid directly to national and subnational organisations and try to ensure those receiving aid have a voice in decisions that affect them (Inter-Agency Standing Committee 2020). However, ‘active’ participation is not always possible or even desired by humanitarian organisations, indicating that ‘desirable’ forms of participation and community organisation are still those set by humanitarian organisations themselves (Chkam, 2016). For example, while humanitarian programmes may strengthen the role of displaced women, they simultaneously constrain the ways these women can participate. Olivius suggested participation serves two main purposes: (i) creating active displaced communities that govern themselves in agreement with humanitarian agency norms and rules, allowing displaced people to feel involved and responsible for camp life; and (ii) fostering capabilities of self-regulation, activity, and responsibility to counter 'dependency syndrome' narratives (Olivius, 2013). Specific groups required targeted support, e.g. providing women vaccinators for girls and including women in agenda setting and project implementation (Jalloh et al*.*, 2020).

Humanitarian action creates parallel responses to national services. Ensuring a strong working relationship between government and humanitarian actors, including NGOs, improves space for action and humanitarian responses. Governments may not readily acknowledge a need for humanitarian assistance, particularly if their position is vulnerable. Humanitarian responses are not always perceived as desirable, for example if agencies change the quality of and accountability for services provision or create expectations (e.g. free services) governments cannot sustain once humanitarian actors have left.

Some findings suggested clear paths to improve health system responses to mass displacement. Cash-transfers appear to increase agency during displacement and positively affect health, with displaced households using cash-transfers appropriately to improve nutrition, health, and education (Versluis, 2014; Ecker et al*.*, 2019). Cash transfers also reduced transactional sex among adolescent girls in Ethiopia (Bermudez et al*.*, 2019). Displaced people are a potential resource in training others to promote better health, e.g. WASH, SRH, and infection prevention. NFE could be used to spread healthy practices and interactive drama sessions may work particularly well with children or poorly-educated adults.

A clear need exists for further coordination and responses integration by all actors at all levels during mass displacement. COVID-19 shows that multisectoral approaches are needed, in which power dynamics between displaced people, humanitarian agencies, and governments are acknowledged and addressed (Gostin et al*.*, 2020). Equity needs to be at the centre of any coherent approach to supporting displaced populations.

### Limitations

This review may have missed some sources due to our search terms. We included publications in all official UN languages plus Hindi so may have missed relevant sources published in other languages. We did not assess source quality as this could have excluded useful findings. Given the relatively large number of sources we could not describe all findings in detail.

## Conclusion

We synthesised existing humanitarian cluster evidence providing lessons for health system responses to mass displacement. This review highlighted the need for coordinated, integrated, and cooperative responses for all levels (international, national, subnational) and actors (e.g. humanitarian system agencies, governments, communities) contextualised to displaced and host population needs and socio-cultural realities.

## Funding

This review is part of an MRC Health Systems Research Initiative Foundation study synthesising evidence from other sectors to strengthen health system responses to mass displacement (MR/S013008/1) awarded to NH. The funder had no involvement in research conduct or manuscript preparation.

## Declaration of Competing Interest

The authors declare that they have no known competing financial interests or personal relationships that could have appeared to influence the work reported in this paper.
